# Supplementary material for: Mechanisms governing the pioneering and redistribution capabilities of the non-classical pioneer PU.1
Source: Nat Commun. 2020 Jan 21;11:402. doi: 10.1038/s41467-019-13960-2 (PMC6972792; doi:10.1038/s41467-019-13960-2)
Supplement: Supplementary file 7 — Source data [file 41467_2019_13960_MOESM7_ESM.zip › Source_Data/FigureS2/FigureS2B_MotifScanOutput/KmeansCluster3/homerResults/motif10.similar.html]

motif10

## Information for motif10

A
G
T
C
G
A
C
T
A
G
C
T
A
G
T
C
G
A
T
C
G
A
C
T
A
G
T
C
G
A
T
C
G
A
C
T
A
G
T
C
G
A
T
C
G
A
C
T
A
G
T
C
G
A
T
C
  
Reverse Opposite:  

C
T
A
G
T
C
A
G
C
T
G
A
C
T
A
G
T
C
A
G
C
T
G
A
C
T
A
G
T
C
A
G
C
T
G
A
C
T
A
G
T
C
A
G
T
C
G
A
C
T
G
A
T
C
A
G
  

|  |  |
| --- | --- |
| p-value: | 1e-109 |
| log p-value: | -2.531e+02 |
| Information Content per bp: | 1.612 |
| Number of Target Sequences with motif | 512.0 |
| Percentage of Target Sequences with motif | 10.74% |
| Number of Background Sequences with motif | 1287.9 |
| Percentage of Background Sequences with motif | 3.04% |
| Average Position of motif in Targets | 99.3 +/- 52.2bp |
| Average Position of motif in Background | 103.5 +/- 94.9bp |
| Strand Bias (log2 ratio + to - strand density) | 0.1 |
| Multiplicity (# of sites on avg that occur together) | 1.35 |
| Motif File: | file (matrix) reverse opposite |

### Similar de novo motifs found

|  |  |  |  |  |  |  |  |
| --- | --- | --- | --- | --- | --- | --- | --- |
| Rank | Match Score | Redundant Motif | P-value | log P-value | % of Targets | % of Background | Motif file |
| 1 | 0.911 | A G C T A G T C G A T C G A C T A T G C G A T C G A C T G A T C G A T C A G C T A G T C G A T C | 1e-62 | -144.269829 | 5.22% | 1.26% | motif file (matrix) |
| 2 | 0.882 | G A C T A G T C G A T C G A C T A G T C G A T C G A C T A G T C G A T C G A C T A G T C G A T C G A C T A G T C | 1e-49 | -114.579389 | 3.21% | 0.59% | motif file (matrix) |
| 3 | 0.826 | A G C T A C G T A G T C A G T C A G C T A G T C G T A C A G C T T G A C G T A C C G A T T A G C A G T C A C G T | 1e-38 | -88.877715 | 2.73% | 0.56% | motif file (matrix) |
| 4 | 0.828 | A G T C G A C T A T G C G T A C G A C T A G T C G A T C G A C T G A T C G A T C G A C T A G C T A G T C | 1e-30 | -69.935019 | 2.79% | 0.74% | motif file (matrix) |
| 5 | 0.810 | C T G A C T A G C T A G C T G A C T A G C T A G C T G A A C T G T C A G C T G A | 1e-24 | -57.466722 | 2.56% | 0.75% | motif file (matrix) |
